# Editorial: Toward an Improved Understanding of Severe and Enduring Anorexia Nervosa

**DOI:** 10.3389/fpsyt.2021.698194

**Published:** 2021-05-28

**Authors:** Phillipa J. Hay, Rebecca J. Park, Stephen W. Touyz

**Affiliations:** ^1^Translational Health Research Institute, Western Sydney University, Sydney, NSW, Australia; ^2^South Western Sydney Local Health District, Camden and Campbelltown Hospitals, Campbelltown, NSW, Australia; ^3^Department of Psychiatry, Warneford Hospital, University of Oxford, Oxford, United Kingdom; ^4^Charles Perkins Centre, InsideOut Institute, University of Sydney, Camperdown, NSW, Australia; ^5^School of Psychology, University of Sydney, Sydney, NSW, Australia

**Keywords:** eating disorder anorexia nervosa, therapy, treatment resistant, longstanding, chronic

This Special Issue presents a collection of 11 articles addressing a range of issues relevant to current dilemmas in the understanding of severe and enduring anorexia nervosa (SEAN). These are the defining characteristics of SEAN, understanding neurobiological, cognitive, and psychosocial mechanisms of illness persistence, and the treatment evidence base.

## Defining Features

Hay and Touyz (1) have posited that there are three defining features of SEAN, namely a persistent state of illness, for at least 3 years, and having had access to at least two evidence based treatments with appropriate person-centered understanding or formulation. In this regard, Dapelo et al. in a trans diagnostic clinical sample reported an investigation of such putative defining features. They tested illness duration against ED symptom severity. Whilst this was not significant, delays in care were however related to illness severity. Illness duration was also not significantly associated with prior incidents of treatment, hospital admissions, medical co-morbidity, or current function. Their findings highlight the importance of adequate access to care and that a diagnosis of SEAN (or SE eating disorder) should not be made where treatment has been insufficient. This point is further made by Gutiérrez and Carrera et al. in their historical review of treatments. They argue that in some instances treatment intractability may rather be treatment ineffectiveness.

Smith and Woodside et al. in investigating treatment intractability as a putative defining feature of SEAN found significant relationships between presence of purging and depressive symptoms, but not eating disorder symptom severity or body weight, with multiple readmissions in a clinical sample of people with anorexia nervosa. Duration of illness was not significantly associated with multiple readmissions, although there was a trend and the sample may have been underpowered. This underscores the premise that all three defining features of SEAN, treatment intractability, severity and duration may occur together but may occur apart and more research is needed to delineate this syndrome or syndromes.

## Understanding Illness Persistence

In exploring symptom persistence Jacquemot and Park et al. in their Mini Review present the literature around altered interception and a novel neuro-circuitry model of its role in the maintenance of symptoms through dysregulation of hunger/satiety cues, interceptive predictive error, negative affect, impaired emotion regulation, and body image disturbance. Het et al. presented preliminary findings showing persisting under-reactive hypothalamus–pituitary–adrenal axis which may present targets for identifying those vulnerable to an eating disorder and possibility thereby of early interventions, preventing the severe and enduring state.

van Elburg et al. pose an “idea to research” in the question is impaired mental capacity, i.e., “the ability to understand and process information on a cognitive and an emotional level” impaired in people with SEAN? If so this could lead to poor decision making and contribute to emotion dysregulation which both may help in understanding persistence of illness and may be remedial with innovative therapies.

Musolino et al. provide a novel socio-anthropological qualitative methodology to understanding the psychological state and symptom meaning making whereby the illness persists. Beyond a sense of loss of identity and sense of self, they found that people may fear symptoms reduction, as losing the illness would impact on their sense of “being-in-the-world” and thus they may experience disembodiment.

## Refining and Developing Treatments

Zhu et al. present a Mini Review of psychological therapy studies for the outpatient care. Whilst highlighting the deficits in evidence they point to several promising approaches that have been tested in participant samples that included a large proportion of people with longstanding illness. These address putative maintaining features such as affective disorder comorbidity, impaired adaptive function and quality of life and suggest merit in refining treatment goals. Clausen et al. ventures into the challenging area of the use of involuntary care. Clausen presents the argument for a more compassionate and judicious use of such legal restraints, allowing the person to negotiate different goals and time frames for recovery.

Tchanturia et al. describe a new approach “Pathway for Eating disorders and Autism developed from Clinical Experience (PEACE)” to integrated care for people with this problematic co-morbidity that likely also contributes to the poor outcomes for this group. Kerr-Gaffney et al. further empirically examined associations within this comorbidity using a cross-sectional network analytic design. The strongest bridging symptoms were low self-confidence and anxiety about social eating and public body exposure. They acknowledged a prospective design is needed to determine the direction of associations.

## Conclusions

This Special Issue has highlighted the urgent need for empirical research testing putative defining features of SEAN and building the evidence base for treatments. Also, people with longstanding AN may not actually be “treatment resistant,” but rather the treatments delivered to them may not have been adequately tailored to meet their needs. Thus, current treatments are ineffective. On a positive note, the papers also provide promise of new treatment targets through an improved understanding of mechanisms of illness persistence.

## Author Contributions

PH conceived and wrote this paper. ST, RP, and PH edited and provided intellectual input to the manuscript. All authors contributed to the article and approved the submitted version.

## Conflict of Interest

PH has received sessional fees and lecture fees from the Australian Medical Council, Therapeutic Guidelines publication, and New South Wales Institute of Psychiatry and royalties/honoraria from Hogrefe and Huber, McGraw Hill Education, and Blackwell Scientific Publications, Biomed Central and Plos Medicine and she has received research grants from the NHMRC and ARC. She is Chair of the National Eating Disorders Collaboration Steering Committee in Australia (2019) and was Member of the ICD-11 Working Group for Eating Disorders (2012–2018) and was Chair Clinical Practice Guidelines Project Working Group (Eating Disorders) of RANZCP (2012–2015). She has prepared a report under contract for Shire Pharmaceuticals in regard to Binge Eating Disorder (BED; July 2017) and Honoraria for training Psychiatrist in BED assessment. ST receives royalties for written work from Hogrefe and Huber, Taylor and Francis, and Routledge. He is the Chair of the Shire/Takeda Clinical Advisory Committee for Binge Eating Disorder, has receive honoraria from Shire/Takeda for commissioned reports, as well as both travel and research grants. He is a consultant to Weight Watchers (WW). ST is a mental health adviser to the Commonwealth (of Australia) Department of Veteran's Affairs. ST is a member of the Commonwealth of Australia's Department of Health Technical Committee for Eating Disorders. He is Editor of the Journal of Eating Disorders. The remaining author declares that the research was conducted in the absence of any commercial or financial relationships that could be construed as a potential conflict of interest.
